# Commentary: Protecting the artery of Adamkiewicz: Highest imperative

**DOI:** 10.1016/j.xjtc.2021.02.006

**Published:** 2021-02-07

**Authors:** Matheus P. Falasa, Salvatore T. Scali, Thomas M. Beaver

**Affiliations:** a General Surgery Residency Program, University of Florida, Gainesville, Fla; b Division of Vascular, Department of Surgery, University of Florida, Gainesville, Fla; c Division of Cardiovascular Surgery, Department of Surgery, University of Florida, Gainesville, Fla

**Figure:**
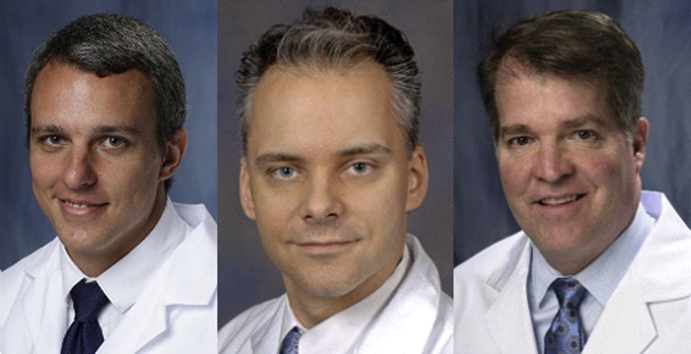
Matheus P. Falasa, MD, Salvatore T. Scali, MD, and Thomas M. Beaver, MD, MPH

**Central Message**

Careful intraoperative management of the artery of Adamkiewicz minimizes the risk of spinal cord ischemia.

See Article page 32.

Plotkin and colleagues^1^ describe a complex case in which a 71-year-old man after prior open thoracoabdominal aortic aneurysm repair presented with an aorto-bronchial fistula and visceral and intercostal pseudoaneurysms. He was treated with a thoracic endograft specifically modified with an intercostal side branch fenestration and subsequent 4-vessel fenestrated endovascular aortic repair. The authors are to be congratulated on their success implementing a novel advanced endovascular technique to the benefit of this patient. Although only applicable to patients with ≥5 mm intercostal arteries, this off-label technique should be feasible at most advanced aortic centers and may further decrease rates of spinal cord ischemia (SCI) after thoracic endovascular aortic repair (TEVAR).

Cerebrospinal fluid drain placement in endovascular aortic aneurysm repair is not without risks. Although at our center we have had minimal morbidity, there are reports of 2% intracranial hemorrhage rate and 2% paraplegia rate secondary to spinal hematoma.^2^ There is a trend in the literature moving away from universal use of prophylactic preoperative spinal fluid drain placement during procedures. A 2019 analysis by the Vascular Quality Initiative found that of 11,473 patients undergoing TEVAR from 2014 to 2019, only 33% underwent routine preoperative spinal drain placement.^3^ A single-center retrospective review of 241 TEVAR procedures found that patients who experienced postoperative SCI were older, had longer intraoperative times, averaged >20 cm aortic coverage, and averaged <2 cm uncovered aorta proximal to the celiac artery, highlighting potential risk factors for SCI.^4^ Prophylactic spinal drainage did not correlate with the development of SCI in this study. Another institution published an experience with a series of 223 patients who underwent TEVAR using a protocol without preoperative spinal drain placement, allowing for permissive hypertension, and focusing on preservation of the left subclavian artery flow, and reported 0% SCI.^5^ Average aortic coverage in this study was 23.0 cm.

Postoperative collateralization of blood flow to the great anterior radiculomedullary artery (also known as the artery of Adamkiewicz) after TEVAR has been studied. In a series of 32 patients, the artery of Adamkiewicz was perfused by a segmental artery distal to the stent graft landing zone in 53%, branches of the left subclavian artery in 33%, and a branch of the external left iliac artery in 13%.^6^ This study highlights the importance of preserving arterial flow and minimizing aortic coverage.

The patient described by Plotkin and colleagues^1^ was at high risk for SCI if the described intercostal artery was covered, regardless of preoperative spinal drainage. This novel endovascular technique represents an exciting development in the mitigation of a dreaded complication.

## References

[1] Plotkin A., Han S.M., Manzur M.F., Cunningham M.J., Fleischman F., Magee G.A. (2021). Intercostal artery incorporation to prevent spinal cord ischemia during total endovascular thoracoabdominal aortic repair. J Thorac Cardiovasc Surg Tech.

[2] Kärkkäinen J.M., Cirillo-Penn N.C., Sen I., Tenorio E.R., Mauermann W.J., Gilkey G.D. (2020). Cerebrospinal fluid drainage complications during first stage and completion fenestrated-branched endovascular aortic repair. J Vasc Surg.

[3] Scali S.T., Giles K.A., Wang G.J., Kubilis P., Neal D., Huber T.S. (2020). National incidence, mortality outcomes, and predictors of spinal cord ischemia after thoracic endovascular aortic repair. J Vasc Surg.

[4] Feezor R.J., Martin T.D., Hess P.J., Daniels M.J., Beaver T.M., Klodell C.T. (2008). Extent of aortic coverage and incidence of spinal cord ischemia after thoracic endovascular aneurysm repair. Ann Thorac Surg.

[5] Weissler E.H., Voigt S.L., Raman V., Jawitz O., Doberne J., Anand J. (2020). Permissive hypertension and collateral revascularization may allow avoidance of cerebrospinal fluid drainage in thoracic endovascular aortic repair. Ann Thorac Surg.

[6] Fukui S., Tanaka H., Kobayashi K., Kajiyama T., Mitsuno M., Yamamura M. (2016). Development of collaterals to the spinal cord after endovascular stent graft repair of thoracic aneurysms. Eur J Vasc Endovasc Surg.

